# Closed-loop stimulation of a delayed neural fields model of parkinsonian STN-GPe network: a theoretical and computational study

**DOI:** 10.3389/fnins.2015.00237

**Published:** 2015-07-10

**Authors:** Georgios Is. Detorakis, Antoine Chaillet, Stéphane Palfi, Suhan Senova

**Affiliations:** ^1^Laboratoire des Signaux et Systèmes, CentraleSupelecGif-sur-Yvette, France; ^2^Faculté des Sciences, Université Paris SudOrsay, France; ^3^AP-HP, Hospital H. Mondor, Service de neurochirurgieCréteil, France; ^4^Institut National de la Santé et de la Recherche Médicale, U955, Equipe 14Créteil, France; ^5^Faculty of Medicine, Université Paris EstCréteil, France

**Keywords:** Parkinson's disease, optogenetics, β-oscillations, closed-loop deep brain stimulation, delayed neural fields, control theory, stability analysis

## Abstract

Several disorders are related to pathological brain oscillations. In the case of Parkinson's disease, sustained low-frequency oscillations (especially in the β-band, 13–30 Hz) correlate with motor symptoms. It is still under debate whether these oscillations are the cause of parkinsonian motor symptoms. The development of techniques enabling selective disruption of these β-oscillations could contribute to the understanding of the underlying mechanisms, and could be exploited for treatments. A particularly appealing technique is Deep Brain Stimulation (DBS). With clinical electrical DBS, electrical currents are delivered at high frequency to a region made of potentially heterogeneous neurons (the subthalamic nucleus (STN) in the case of Parkinson's disease). Even more appealing is DBS with optogenetics, which is until now a preclinical method using both gene transfer and deep brain light delivery and enabling neuromodulation at the scale of one given neural network. In this work, we rely on delayed neural fields models of STN and the external Globus Pallidus (GPe) to develop, theoretically validate and test *in silico* a closed-loop stimulation strategy to disrupt these sustained oscillations with optogenetics. First, we rely on tools from control theory to provide theoretical conditions under which sustained oscillations can be attenuated by a closed-loop stimulation proportional to the measured activity of STN. Second, based on this theoretical framework, we show numerically that the proposed closed-loop stimulation efficiently attenuates sustained oscillations, even in the case when the photosensitization effectively affects only 50% of STN neurons. We also show through simulations that oscillations disruption can be achieved when the same light source is used for the whole STN population. We finally test the robustness of the proposed strategy to possible acquisition and processing delays, as well as parameters uncertainty.

## 1. Introduction

Pathological conditions in Parkinson's disease, Huntington's disease or epileptic seizures are often associated with abnormal oscillations in specific brain areas (Bragin et al., 2010; Devergnas and Wichmann, 2014). In particular, parkinsonian motor symptoms result from a dopamine depletion in the striatum and strong neural synchronization, bursting and sustained oscillations in low frequencies can be observed (Bergman et al., 1994; Brown et al., 2001; Jenkinson and Brown, 2011). This oscillatory activity is especially detectable in global signals such as local field potentials (LFPs) (Stein and Bar-Gad, 2013). The precise relation between these sustained oscillations and motor symptoms is still a matter of debate. Nonetheless, there is clear evidence that low-frequencies activity in the LFP of the subthalamic nucleus (STN) is correlated to parkinsonian motor deficit. More precisely, bradykinesia and rigidity are related to strong and sustained activity in β-frequencies (13–30 Hz) (Kühn et al., 2006; Hammond et al., 2007; Little et al., 2012), while low γ-frequencies (31–45 Hz) correlate with tremor severity (Beudel et al., 2015). Sustained β-activity is not reported in healthy conditions (Bar-Gad et al., 2003; Heimer et al., 2006; Schwab et al., 2013). Among the possible hypotheses for the mechanisms underlying this pathological synchronization onset, a possible pacemaker role of the network formed by the excitatory STN and the inhibitory external globus pallidus (GPe) has been proposed based on *in vitro* experiments (Plenz and Kital, 1999). With striatal origins and cortical STN patterning, this role of the STN-GPe as a pacemaker is one of the main hypotheses for this pathological oscillations onset (Bevan et al., 2002; Lang and Zadikoff, 2005). Experiments conducted on non-human primate models of Parkinson's disease confirm the importance of reciprocal STN-GPe connections on oscillatory activity onset (Nambu and Tachibana, 2014). Although not formally proven *in vivo*, this pacemaker effect of the STN-GPe network is supported by mathematical models, fitted to neurophysiological data, which effectively reproduce sustained oscillations in parkinsonian conditions (Terman et al., 2002; Holgado et al., 2010; Merrison et al., 2013; Pasillas-Lépine, 2013). In order to test this hypothesis, there is a critical need for developing theoretical strategies of neuromodulation that could be implemented experimentally in order to control sustained oscillations in the STN.

Deep Brain Stimulation (DBS, Benabid et al., 1991) is a neuromodulation method that consists in chronically implanting electrodes in specific targeted brain areas and in applying electrical currents to alleviate neurological symptoms (Lozano and Lipsman, 2013). In the case of Parkinson's disease, three different targets (thalamus, globus pallidus pars internalis and subthalamic nucleus) have been identified to reduce motor symptoms such as tremor or/and rigidity, akinesia, the STN being the most prescribed. Although the electrical DBS's mechanisms of action are still a matter of debate, it has been suggested that DBS of the STN may affect β-synchrony in the targeted neural population (Eusebio et al., 2011). In most existing DBS treatments, the electrical signal is delivered in an open-loop manner, meaning regardless of the patient's state or brain activity. As reviewed in Carron et al. (2013), several attempts have recently been made to optimize DBS by taking into account some measurements of the patient brain activity. These include adaptive and on-demand stimulation (Marceglia et al., 2007; Graupe et al., 2010; Rosin et al., 2011; Santaniello et al., 2011; Little et al., 2013), delayed and multi-site stimulation (Omel chenko et al., 2008; Batista et al., 2010; Pfister and Tass, 2010; Lysyansky et al., 2011; Tass et al., 2012), optimal control strategies (Feng et al., 2007), and closed-loop firing rate regulation (Wagenaar et al., 2005; Luo et al., 2009; Franci et al., 2011; Pasillas-Lépine et al., 2013). Another strategy, not included in that review, is Schiff (2010), which advocates for the use of Kalman filtering to enhance DBS strategies. The review Carron et al. (2013) underlines a clear gap between model-based closed-loop DBS strategies, whose efficiency is most of the time assessed *in silico*, and the DBS signals actually tested *in vivo*.

Optogenetics is a promising tool to unravel pathological oscillations onset mechanisms and to explore closed-loop strategies to optimize DBS treatment. Using a viral vector, genes coding for neuronal membrane ionic channels, sensitive to precise wavelengths, are selectively transferred to precisely targeted neurons, which become photosensitive. Thus, simple pulses of intense light, e.g., through implanted optical fibers, can induce the response of the photosensitized neurons (Boyden et al., 2005; Pastrana, 2010). By contrast with electrostimulation, optical neuromodulation does not induce any stimulation artifacts on electrical recordings. It also allows sharpest targeting of specific neuronal subpopulations. In addition, the development of bi-color optogenetics stimulation and bi-switchable opsins such as Halo-ChR2 (Han and Boyden, 2007), now allows for simultaneous excitation and inhibition of the neural population in a controlled way, whereas electrical DBS may perform both excitation and inhibition on different compartments of the same neurons or on different neurons depending on their orientations relative to the electrical field (Ranck, 1975; McIntyre et al., 2004). Thus, combined with electrode measurements, optogenetics may offer unprecedented possibilities to achieve closed-loop stimulation of neural structures, in particular for counteracting pathological oscillations in animal models (Chaillet et al., 2014). First experimental attempts include those described by Paz et al. (2012), where closed-loop optical stimulation was used on rats in order to interrupt seizures after cortical injury.

A particular way to avoid the generation of sustained oscillations in a parkinsonian STN-GPe network is to artificially reduce the gain of the STN-GPe loop. This strategy has been developed in Pasillas-Lépine et al. (2013)^1^ using tools from linear control theory. That theoretical paper demonstrates that, by delivering a photostimulation whose frequency is proportional to the measured unitary activity, sustained oscillations are impeded in the parkinsonian basal ganglia model proposed in Holgado et al. (2010). It further proposes a filtering strategy to cope with inherent acquisition and processing delays. In view of its simplicity and ease of technological implantation, this closed-loop DBS strategy is very appealing. Nonetheless, this encouraging result still suffers from important limitations. The main one stands in the employed model, which summarizes the activity of each neural population by a single averaged variable and thus fails at taking into account spatial heterogeneity within STN and GPe. The first aim of this theoretical paper is to propose a model of the parkinsonian STN-GPe network, able to take into account both temporal and spatial evolutions of the activity.

Another limitation of the study conducted in Pasillas-Lépine et al. (2013) is that it relies on a linearized model, and thus neglects the inherent nonlinear effects arising from the neurons f-I curves and the saturation of the stimulation signal. The works Faye and Faugeras (2010); Veltz and Faugeras (2011) provide a relevant mathematical framework to study the proposed spatiotemporal model analytically, despite the combined effects of delays and nonlinearities. The second objective of this paper is to propose a closed-loop stimulation strategy enabling disruption of sustained oscillations in this spatiotemporal model of the STN-GPe network, and to prove its efficiency both mathematically and numerically. This stimulation signal is designed to be realistically implementable in order to test the role of sustained oscillations in parkinsonian symptoms in future experiments.

The third objective of this paper is to validate both the model and the associated closed-loop stimulation signal based on *in silico* experiments that take into account specific experimental constraints that would occur in an *in vivo* testing. These constraints include parameter uncertainties, imperfect photosensitization or insufficient illumination of STN neurons, use of a single light source for the whole STN population, and unavoidable acquisition and processing delays.

## 2. Materials and methods

### 2.1. Neural fields model of the STN-GPe network

In parkinsonian conditions, pathological oscillations are commonly observed in the dorsolateral region of the STN (Zaidel et al., 2010). We focus on this specific area and on the GPe neurons that either project or receive projections from that region. From now on, we will simply refer to these regions as STN and GPe for simplicity. A finer study of the pathological oscillations onset in the parkinsonian STN-GPe network requires to include the evolution of their activity in both time and space. To this aim, we model the STN and GPe populations as delayed neural fields (Coombes, 2005; Bressloff, 2012). Indicating the STN and GPe populations with indexes 1 and 2 respectively, the STN-GPe network is thus modeled as coupled delayed neural fields:

(1a) $$\begin{array}{l} \tau_{1}\frac{\partial z_{1}}{\partial t}(r,t)=-z_{1}(r,t)\\ +S_{1}\left(\sum_{j=1}^{2}\int_{\Omega }w_{1j}(r,r^{\prime })z_{j}(r^{\prime },t-d_{j}(r,r^{\prime }))dr^{\prime }+I_{1}^{\text{ext}}(r,t)\right) \end{array}$$

(1b) $$\begin{array}{l} \tau_{2}\frac{\partial z_{2}}{\partial t}(r,t)=-z_{2}(r,t)\\ +S_{2}\left(\sum_{j=1}^{2}\int_{\Omega }w_{2j}(r,r^{\prime })z_{j}(r^{\prime },t-d_{j}(r,r^{\prime }))dr^{\prime }\;+\;I_{2}^{\text{ext}}(r,t)\right). \end{array}$$

**Figure 1:**
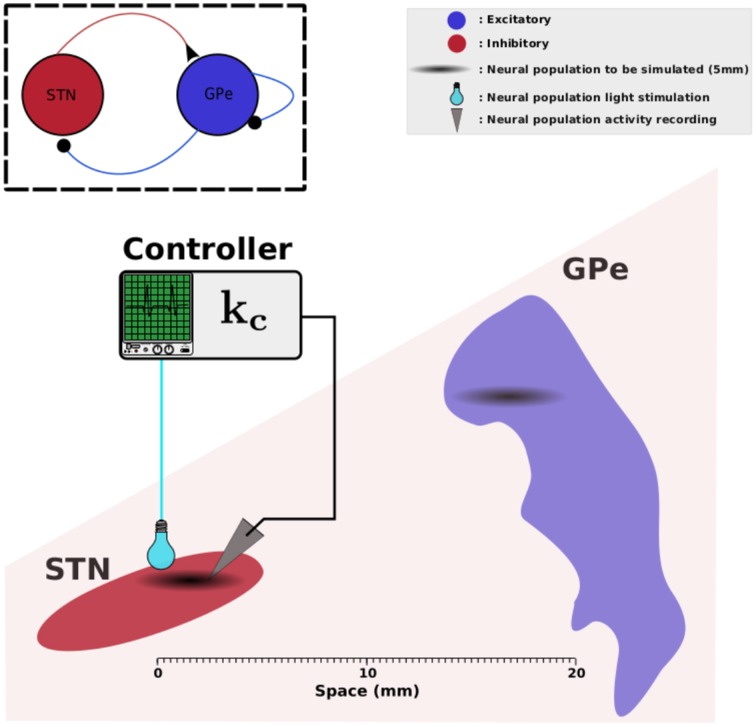
**STN-GPe network**. This figure illustrates the physical arrangement of STN and GPe. We assume that the zones involved in pathological oscillations in STN and GPe measure 2.5 mm each (Zaidel et al., 2010); they are indicated in black clouds. The light-green box illustrates a controller that reads data from STN and applies a photostimulation to the same neural population. Elements of this figure have been found in Wikipedia and they are licensed according to CC (http://creativecommons.org/licenses/by-sa/3.0).

*z*_1_(*r*, *t*) and *z*_2_(*r*, *t*) represent the neuronal activity of the neurons at position *r* and at time *t* for STN and GPe respectively. Ω denotes a compact set of ℝ^*p*^, *p* ∈ {1,2,3}, representing the physical support of the network neuronal populations. τ_1_ and τ_2_ are the time constant STN and GPe activities. *w_ij_*(*r*, *r*') describes the synaptic strength between location *r*' in population *j* and location *r* in population *i*. *d*_1_(*r*, *r*') and *d*_2_(*r*, *r*') represent the axonal time delays between a pre-synaptic neuron at position *r*' and a post-synaptic neuron at position *r*. *S*_1_ and *S*_2_ are the activation functions of STN and GPe respectively. *I*_1_^ext^(*r*, *t*) and *I*_2_^ext^(*r*, *t*) account for the external inputs of STN and GPe. In this work, we consider that the STN neurons expresses the gene that codes for photoactivatable proteins and receive sufficient illumination from the stimulation device. In other words, the STN activity can be modulated with light stimulation. *I*_1_^*ext*^(*r*, *t*) thus decomposes into a stimulation signal *u*(*r*, *t*) that we can regulate and the inputs *I*_1_(*r*, *t*) from other cerebral structures, especially cortical inputs. The GPe is not directly affected by the stimulation and hence sums up to inputs *I*_2_(*r*, *t*) from other brain areas, specifically from the striatum. The overall setup is depicted by Figure 1.

### 2.2. Closed-loop stimulation signal

As will be seen in Section 3, the STN-GPe model (Equation 1) may exhibit sustained oscillations even when the exogenous inputs *I*_1_^*ext*^ and *I*_2_^*ext*^ are constant in time. These oscillations result from the combined effects of transmission delays and too strong synaptic weights between STN and GPe. As originally proposed in Holgado et al. (2010), these increased synaptic gains in pathological conditions may result from dopamine depletion. In order to attenuate these pathological oscillations, we rely on a proportional stimulation law, in the spirit of Pasillas-Lépine et al. (2013). Proportional control constitutes the basics of control theory; it consists in applying an input which is proportional to the difference between the measured state and a targeted reference:

(2) $$u(r,t)=-k_{c}\left(z_{1}(r,t)-z_{ref}(r)\right),$$

where *k_c_* is a positive control gain that can be adjusted at will, and *z_ref_*(*r*) represents a prescribed rate for the STN population. The stimulation signal is thus assumed to act as an external rate on the STN. It may act as an either inhibitory or excitatory input, depending on the sign of *u*(*r*, *t*). This is in agreement with the recent development of bi-color optogenetics stimulation and bi-switchable opsins such as Halo-ChR2 (Han and Boyden, 2007), which enables both excitation and inhibition of the same neuronal population.

The rationale behind this proportional closed-loop stimulation is rather intuitive: when the measured STN activity *z*_1_(*r*, *t*) overpasses the prescribed rate *z_ref_*(*r*), the stimulation tends to decrease the STN activity, translating in a negative sign of the stimulation signal *u*(*r*, *t*). On the contrary, when the measured STN activity is too low compared to the target rate, the stimulation excites the STN (*u*(*r*, *t*) comes with a positive sign). In both cases, the amplitude of the delivered stimulation signal is proportional to the difference between the STN measured activity and the prescribed one. Note that the implementation of this closed-loop stimulation signal requires the real-time measurement of the STN activity. No measurements on the GPe or other brain areas are needed.

Plugging the closed-loop stimulation signal (Equation 2) into the spatiotemporal model (Equation 1) leads to the following dynamics:

(3a) $$\begin{array}{l} \tau_{1}\frac{\partial z_{1}}{\partial t}=-z_{1}+S_{1}(\sum_{j=1}^{2}\int_{\Omega }w_{ij}(r,r^{\prime })z_{j}(r^{\prime },t-d_{j}(r,r^{\prime }))dr^{\prime }\\ \;+\;I_{1}(r,t)-k_{c}\alpha (r)\left(z_{1}(r,t)-z_{ref}(r)\right)) \end{array}$$

(3b) $$\begin{array}{l} \tau_{2}\frac{\partial z_{2}}{\partial t}=-z_{2}+S_{2}(\sum_{j=1}^{2}\int_{\Omega }w_{ij}(r,r^{\prime })z_{j}(r^{\prime },t-d_{j}(r,r^{\prime }))dr^{\prime }\\ \;+\;I_{2}(r,t)). \end{array}$$

α(*r*) ≥ 0 is a position-dependent gain accounting for the strength of the stimulation influence at position *r*. It typically decreases as a function of the distance to the stimulation device, due to light absorption in brain tissues (Deng et al., 2014). Its value may also be affected by the quality of photosensitization of the targeted neurons. In the case when photosensitization is not effective or light stimulation is not received, it holds that α(*r*) = 0.

### 2.3. Tools to assess performance and robustness

In order to numerically investigate the effects of the proposed closed-loop DBS method described above, we propose five *in silico* experimental protocols.

- Protocol A tests the ability of the proposed spatiotemporal model to generate sustained oscillations in the β band. This test is conducted both on a nominal set of parameters deduced from data available in the literature (see Section 2.4) and on variations around these nominal values.
- Protocol B assesses the efficiency of the proposed closed-loop stimulation signal to disrupt these sustained oscillations.
- Protocol C studies the performance of the closed-loop stimulation signals in case of poor STN photosensitization.
- Protocol D tests the implementability of the closed-loop stimulation policy when a single light source is available for the whole STN population.
- Protocol E investigates how acquisition and processing delays may affect the performance of the closed-loop stimulation signal.

### 2.4. Parameters selection

For simplicity and clarity of exposition, we consider only 1-dimensional spatial distributions: Ω is picked as the interval [0, 15]mm, as the STN and GPe are both contained in zone of length 15mm in adult human brain. The STN activity lies in the subregion Ω_1_ = [0, 2.5]mm, while the GPe stands in the subregion Ω_2_ = [12.5, 15] mm. All these values are in accordance with adult human brain physiology (Mai et al., 1997).

An advanced identification of parkinsonian activity of STN and GPe rate dynamics has been performed in Holgado et al. (2010) based on experimental data available in the literature. We partly rely on that work to identify some parameters of the model. More precisely, in that reference, the time constants τ_1_ and τ_2_ were taken as the membrane time constants of the neurons involved and can be taken as 6 ms and 14 ms according to e.g., Paz et al. (2005) and Kita and Kitai (1991) respectively. The activation functions *S*_1_ and *S*_2_ were fitted to experimental data available in Kita and Kitai (1991) and Deister et al. (2009) among others. They were taken as sigmoidal functions:

$$S_{i}(x)=\frac{m_{i}b_{i}}{b_{i}+(m_{i}-b_{i})e^{-4x/m_{i}}},$$

where *m*_1_ = 300 spk/s, *b*_1_ = 17 spk/s, *m*_2_ = 400 spk/s and *b*_2_ = 75 spk/s. It can easily be checked that the maximum slopes of these functions are ℓ_1_ = ℓ_2_ = 1.

The remaining parameters cannot be readily taken from Holgado et al. (2010) as they are space-dependent, while Holgado et al. (2010) relied on an averaged model. The axonal delays *d*_*i*_(*r*, *r*') are derived based on the physical distance between *r* and *r*' and the velocity *c*_*i*_ of spike propagation along the axons under concern:

$$d_{j}(r,r^{\prime })=\frac{\left|r-r^{\prime }\right|}{c_{j}}.$$

We picked *c*_1_ = 2.5 m/s and *c*_2_ = 1.4 m/s, in accordance with Kita et al. (1983). The inputs *I*_1_(*r*, *t*) and *I*_2_(*r*, *t*) encompass activities from other brain areas, mainly from cortex for STN and from striatum for GPe. Although they were assumed constant in time in our theoretical analysis (see Section 3.1), we have taken them as white noises with variance 0.05 centered at 27 spk/s for cortex and 2 spk/s for striatum (the values have been taken from Holgado et al., 2010). Also in line with Holgado et al. (2010), these input rates were multiplied by uniform synaptic weights of 12.5 (cortical input to STN) and 110 (striatal input to GPe). The addition of a noise to these constant inputs makes the cortical and striatal influences more physiologically plausible and in turn allows to test the robustness of the proposed closed-loop stimulation strategy. The striatal influence on GPe being mostly inhibitory (Kita, 2007), *I*_2_(*r*, *t*) was taken with a minus sign.

**Figure 2:**
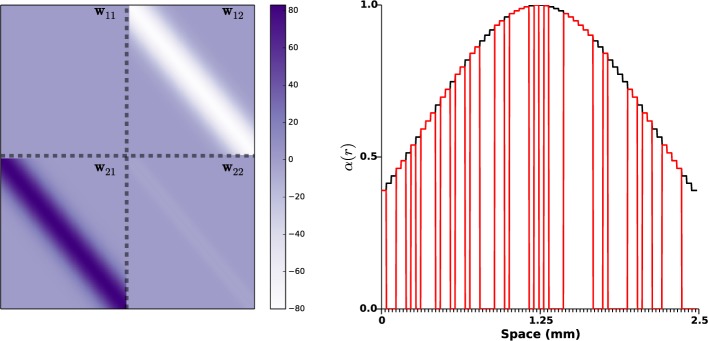
**Synaptic weights distributions and photosensitization distribution**. On the left side panel, the distributions *w_ij_* of the synaptic strengths are represented. The gray dashed lines indicate the space between STN and GPe (10 mm in an adult human brain, Mai et al., 1997). Right panel shows a realization of function α(*r*), the black curve indicates a STN photosensitization of 100%, the red line corresponds to a random photosensitization of 50%.

The kernel *w*_11_ accounting for synaptic coupling within STN was taken as zero since there is no evidence of self-excitatory STN connections (Marani et al., 2008). The other kernels, modeling synaptic weights between the populations (*w*_12_ and *w*_21_) or within the GPe (*w*_22_), were taken as Gaussian functions as represented by Figure 2 (left panel). Synaptic strengths *w*_12_ and *w*_21_ follow the principle of short-range excitation/inhibition. This implies that if a neuron of STN project to a neuron of GPe, the former excites the neighbor neurons in GPe as well. More precisely, *w*_12_ and *w*_21_ were taken as^2^

$$\begin{array}{l} w_{12}(r,r^{\prime })=-g_{12}(|r-r^{\prime }-\mu_{2}|),\;\forall r\in \Omega_{1},r^{\prime }\in \Omega_{2}\\ w_{21}(r,r^{\prime })=g_{21}(|r-r^{\prime }-\mu_{1}|),\;\forall r\in \Omega_{2},r^{\prime }\in \Omega_{1}\\ w_{22}(r,r^{\prime })=-|r-r^{\prime }|g_{22}(|r-r^{\prime }|),\;\forall r,r^{\prime }\in \Omega_{2}, \end{array}$$

where μ_1_ = 1.25mm and μ_2_ = 13.25mm indicate the centers of STN and GPe respectively, and the Gaussian functions *g*_*ij*_ are defined as

$$g_{ij}(x):=K_{ij}\exp\left(-\frac{x^{2}}{2\sigma_{ij}}\right).$$

The variance σ_12_ and σ_21_ were taken identical (0.03 mm^2^) based on the numerical and computational studies (Terman et al., 2002; Park et al., 2011), which indicate that STN and GPe neurons are connected to a small portion of GPe and STN neurons respectively. The variance σ_22_ within GPe is taken smaller (σ_22_ = 0.015) as GPe lateral projections are more local (François et al., 1984). The amplitudes *K_ij_* of the synaptic weights distribution were picked in order for the mean activity to fit the simulations presented in Holgado et al. (2010): *K*_12_ = 30, *K*_21_ = 38, and *K*_22_ = 2.55. Note that the amplitude of *w*_22_ is much lower than the other two, as the number of lateral connections within GPe is small (Sadek et al., 2005). *w*_12_ and *w*_22_ were taken negative as the projections from GPe neurons are mostly GABAergic (Sato et al., 2000a); on the other hand *w*_21_ was picked positive, as STN projections are mostly glutamatergic (Sato et al., 2000b). Note that, although this has little influence on the simulations result, *w*_22_ is not a pure Gaussian function: it is zero when *r* = *r*'. This was done in order to provide little synaptic coupling with GPe neurons in the immediate vicinity as little correlations have been observed between neighboring neurons in the GPe (Bar-Gad et al., 2003). This parameters choice lead to a *L*_2_-norm of lateral GPe connections ℓ_2_||*w*_22_|| = 1 × 0.95 < 1, thus fulfilling the requirement (Equation 4) of our theoretical result. This rather small value can be interpreted as the absence of endogenous oscillations within GPe (see Section 3.1 for details). In line with e.g., Plenz and Kital (1999); Nambu and Tachibana (2014), the observed pathological oscillations do not find their roots within GPe, but result from interactions between neuronal populations.

Finally, the function α(*r*) accounting for the impact of light stimulation was taken as a Gaussian function (zero outside Ω_1_) of amplitude 1, centered at the middle of the STN population (1.25mm), and variance 1.25 mm^2^: see right panel of Figure 2. This choice corresponds to a photo-sensitization of all STN neurons and takes into account light absorption in the medium (as the central STN neurons are more impacted by stimulation than neurons in the periphery).

The parameters used in each *in silico* protocols are summarized in Table 1.

**Table 1:** **Model parameters in each protocol**.

**Protocol**	***K*_12_**	**σ_12_**	***K*_21_**	**σ_21_**	***K*_22_**	**σ_22_**	**τ_1_**	**τ_2_**	***c*_1_**	***c*_2_**	***k_c_***
(A)	30 ± 35%	0.03	38± 35%	0.03	2.55± 35%	0.015	6	14	2.5± 35%	1.4± 35%	0
(B),(C),(E)	30	0.03	38	0.03	2.55	0.015	6	14	2.5	1.4	2
(D)	30	0.03	38	0.03	2.55	0.015	6	14	2.5	1.4	6.5

*K_ij_ and σ_ij_ are the amplitude and variance of the synaptic weights w_ij_, τ_1_, and τ_2_ are the time constants of STN and GPe respectively (ms), c_1_ and c_2_ are the axonal propagation velocities from STN and GPe respectively (mm/ms). k_c_ is the gain of proportional closed-loop stimulation. Protocol A: Sensitivity analysis, Protocol B: Disruption of pathological oscillations, Protocol C: Poor STN photosensitization, Protocol D: Single light source, Protocol E: Effect of acquisition and processing delays*.

### 2.5. Simulation details

In order to simulate the delayed neural fields described by Equation (3), we normalized the spatial domain to Ω_*si*m_ = [0, 1]. All synaptic strengths and delays are computed according to this normalized interval. We discretized Ω_*sim*_ using *m* spatial nodes representing unitary subregions of the populations: *m*/6 for the STN, *m*/6 for the GPe and the rest for silent neurons modeling the physical distance between STN and GPe. The temporal integration of the system has been done by the Forward Euler method. All the simulation parameters are gathered in Table 2.

**Table 2:** **Simulation parameters**.

**Protocol**	***t_f_***	***dt***	***m***	**Ω_*sim*_**	***c*_1_**	***c*_2_**	***T***
(A)	1000	1.0	60	[0, 1]	0.166 ± 35%	0.09 ± 35%	0
(B),(C),(D)	1000	1.0	60	[0, 1]	0.166	0.09	0
(E)	1000	1.0	60	[0, 1]	0.166	0.09	0-20

*t_f_ is the total simulation time (ms), dt is Euler's method time step (ms), m is the number of spatial discretization units, Ω_sim_ is the normalized spatial interval (physical interval: Ω = [0, 15] mm), c_1_ and c_2_ are the normalized axonal propagation velocities of action potentials from STN and GPe respectively (mm/ms), T is the acquisition and processing delays for the real-time estimation of STN activity (ms). Protocol A: Sensitivity analysis, Protocol B: Disruption of pathological oscillations, Protocol C: Poor STN photosensitization, Protocol D: Single light source, Protocol E: Effect of acquisition and processing delays*.

We run all the simulations on a Dell Desktop Machine equipped with an Intel i5 processor and 8 GB memory. The average running time of a simulation was 0.7 s. All the simulations are written in Python (Numpy, Scipy, Matplotlib). The source code is freely distributed under the GPL License and can be found on-line at Github^3^.

## 3. Results

### 3.1. Provable disruption of pathological oscillations

Our main theoretical result states that the delayed neural fields (Equation 3) can always be stabilized by the proportional closed-loop stimulation (Equation 2), provided that the GPe does not exhibit endogenous sustained oscillations. More precisely, we have the following result, whose proof is provided in Appendix.

Consider any admissible target pattern *z_ref_*(*r*). Let the activation functions *S*_1_ and *S*_2_ be bounded, increasing, and with maximum slope ℓ_1_ and ℓ_2_ respectively. Assume that the lateral synaptic weights in the GPe satisfy

(4) $$\sqrt{\int_{\Omega }\int_{\Omega }w_{22}{\left(r,r^{\prime }\right)}^{2}dr^{\prime }dr}<\frac{1}{\ell_{2}},$$

and that the photosensitization is efficient over all STN, meaning that α(*r*) > 0 for all *r* ∈ Ω. Assume further that the inputs *I*_1_(*r*, *t*) and *I*_2_(*r*, *t*) from other brain areas are constant in time. Then, for any feedback gain *k_c_* sufficiently large, the neural fields under closed-loop stimulation (Equation 2) is stable and, from almost all admissible initial conditions^4^, the STN and GPe activities exponentially converge to constant patterns.

Condition (Equation 4) requires a sufficiently small *L*_2_-norm for the lateral synaptic weights *w*_22_ of the GPe. It has a physical interpretation: it imposes that the *internal* dynamics of the GPe should not exhibit instability (see e.g., Faye and Faugeras, 2010). Thus, proportional closed-loop stimulation can compensate for oscillations generated either within the STN (large *w*_11_) or due to too strong interactions between the STN and GPe (large *w*_12_ or *w*_21_), but might be insufficient to tackle endogenous oscillations within the GPe (large *w*_22_).

### 3.2. Protocol A: sensitivity analysis

**Figure 3:**
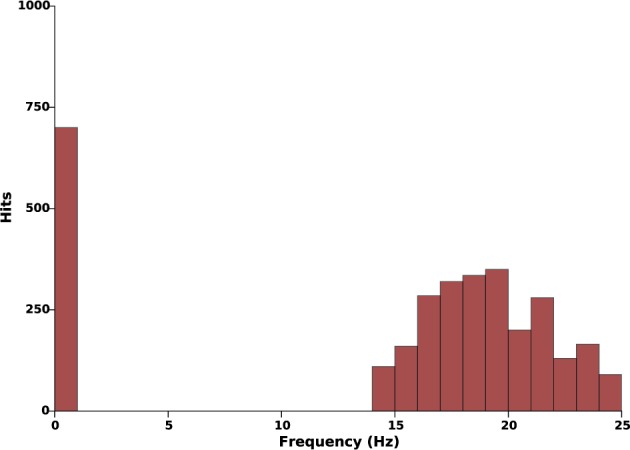
**Effects of parameters uncertainty on the frequency of sustained oscillations**. This histogram illustrates the occurrences of different oscillations frequencies when the parameters *K*_12_, *K*_21_, *K*_22_, *c*_1_, and *c*_2_ evolve in a range of ±35% their nominal values (see Tables 1, 2). In all 10^5^ simulations, when oscillations take place, they lie within the β-band.

We start by assessing the capability of the proposed model (Equation 3) to robustly produce sustained oscillations with the parameters choice described in Section 2.4. Tight parameters identification is usually incompatible with experimental constraints: the robustness of the model is therefore a crucial necessity. To this aim, we systematically modified the amplitude of the synaptic weights distribution (*K*_12_, *K*_21_, and *K*_22_) and the axonal transmission velocities (*c*_1_ and *c*_2_) in ranges of plus or minus 35% their nominal values. Ten values in this range were tested for each parameter, thus corresponding to a total of 10^5^ simulations. No stimulation was applied in this protocol (*k_c_* = 0). For each simulation, we estimated the main harmonic of the STN and GPe activity, as the maximum peak of the Fourier transform of the signals after transients. The result is summarized by a histogram that illustrates the different oscillations frequencies that can be generated by the model: see Figure 3.

This histogram suggests reasonable robustness to parameter uncertainty as, in all simulations, the main oscillations harmonic either lied within 13–25 Hz or was zero. In other words, in these parameters ranges, oscillations either take place in the β-band or do not take place at all.

**Figure 4:**
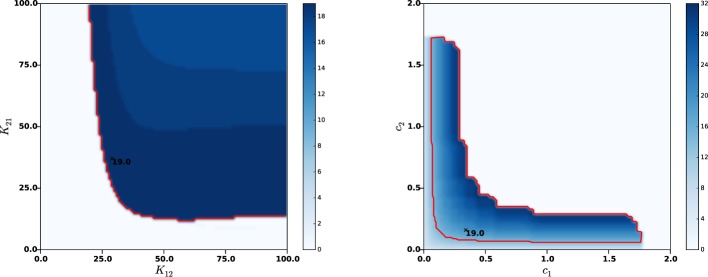
**Effects of synaptic strengths and axonal velocities on the frequency of oscillations**. Left panel: main harmonic frequency of STN and GPe oscillations as a function of the synaptic distributions amplitudes *K*_12_ and *K*_21_ between STN and GPe (left panel) and as a function of the axonal propagation delays *c*_1_ and *c*_2_ (right panel). The red contour indicates β-band frequencies. The nominal values of the parameters are indicated with a black cross, together with the corresponding oscillations frequency.

In order to assess more tightly the role of each parameters in the sustained oscillations onset, we estimated their frequency by varying only two parameters in a wider range, while leaving all other parameters to their nominal values. It appeared that the amplitude *K*_22_ of lateral GPe synaptic weights may result in sustained oscillations or constant activity, but has little effect on the oscillations frequencies (curves not reported). On the contrary, the synaptic weights between STN and GPe (*K*_12_ and *K*_22_) and the axonal velocities (*c*_1_ and *c*_2_) do play a crucial role in the frequency of sustained oscillations. Figure 4 shows the frequencies as a function of *K*_12_ and *K*_21_ (left panel) and as a function of *c*_1_ and *c*_2_ (right panel).

It can be observed that each of the considered parameters can be significantly modified without compromising the β nature of sustained oscillations. Nonetheless, too low synaptic weights or two high axonal velocities (meaning lower transmission delays between STN and GPe) may compromise the very oscillations onset. This is in agreement with the analyses conducted on an averaged rate model in Holgado et al. (2010) and Pasillas-Lépine (2013), which clearly showed that sustained oscillations results from the combined effects of two factors: too strong synaptic coupling between STN and GPe resulting from dopamine depletion and axonal propagation delays. This is also in agreement with the basics of control theory, which predicts that instability is favored by high coupling and delays.

### 3.3. Protocol B: sustained oscillations disruption

**Figure 5:**
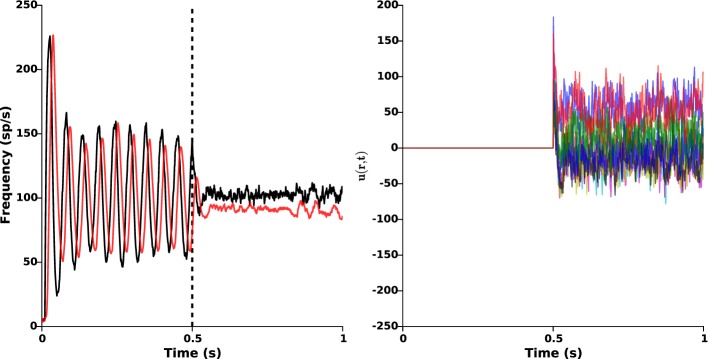
**Oscillations disruption**. The left panel illustrates the disruption of pathological β-oscillations by applying the closed-loop stimulation signal with gain *k_c_* = 2 and target reference *z_ref_*(*r*) = 0 from time *t* = 0.5 s. Red and black curves indicate the time evolution of the GPe and STN mean activities, respectively. Right panel shows the evolution of the control signal *u*(*r*, *t*) = −*k_c_*(*z*_1_(*r*, *t*) − *z_ref_*(*r*)).

As seen in the above section, the nominal parameters lead to sustained β-oscillations (approximately 19 Hz) in both STN and GPe. In order to counteract these pathological oscillations, we apply the proposed closed-loop stimulation (Equation 2) from time *t* = 0.5 s. The results are presented in Figure 5, which clearly shows oscillations disruption. Once stimulation is ON, the remaining oscillations mostly result from the cortical and the striatal stochastic inputs (*I*_1_(*r*, *t*) and *I*_2_(*r*, *t*)).

### 3.4. Protocol C: poor STN photosensitization

**Figure 6:**
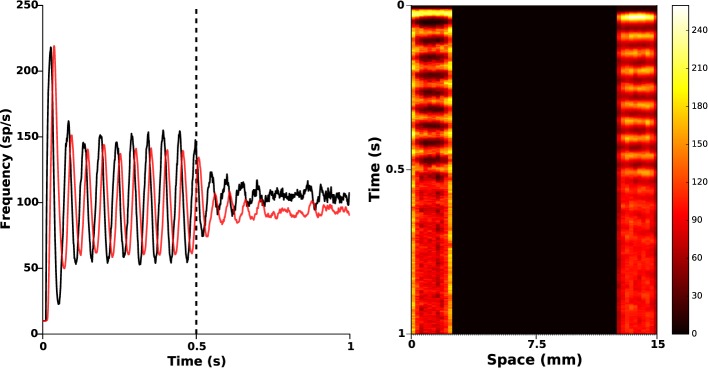
**Poor STN photosensitization**. 50% of the function α(*r*) was randomly silenced. Closed-loop stimulation is applied from time *t* = 0.5 s with gain *k_c_* = 2 and target reference *z_ref_*(*r*) = 0. **Left:** red and black curves illustrate time evolution of the mean activities of GPe and STN, respectively. It is apparent that the attenuation is not as successful as in Figure 5, but oscillations are still attenuated. **Right:** spatiotemporal evolution of the activity in STN (left) and GPe (right).

Depending on the efficacy of transgenesis linked to the viral vector itself, its envelope, its promoter, and to the targeted neurons, it may happen that photosensitization does not affect all STN neurons. It is important to check whether the closed-loop stimulation signal still succeeds in disrupting pathological oscillations. To that end, we randomly put to zero 50% of the values of α(*r*), corresponding to a photosensitization of only half of the STN population (see Figure 2, right panel), while keeping the same value of the feedback gain (*k_c_* = 2). The results are presented in Figure 6. They indicate that the proposed closed-loop stimulation signal is robust to poor photosensitization, as the oscillations are still reduced although less efficiently than when all STN cells are successfully photosensitized.

**Figure 7:**
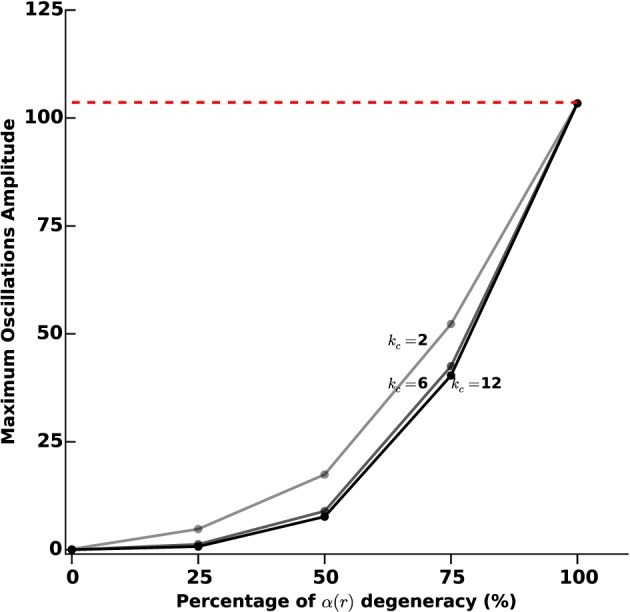
**Sustained oscillations amplitude vs. photosensitization degeneracy**. We measure the efficiency of the proposed closed-loop stimulation through the amplitude of the remaining oscillations. We consider an increasing degeneracy of α(*r*), corresponding to an increasing number of STN neurons that do not respond to photostimulation. The three curves correspond to different values of the feedback gain *k_c_* (2, 6, and 12). The darkest curve corresponds to the highest value and the lighter one to the lowest value. The red dashed line indicates the oscillations maximum amplitude when no stimulation is applied.

In order to further investigate the link between the level of photosensitization of STN neurons and the efficacy of the proposed closed-loop stimulation strategy, we plot the maximum amplitude of observed β-oscillations as a function of the α(*r*) degeneracy (meaning the percentage of STN cells that do not respond to light stimulation). The results are presented in Figure 7, for various values of the percentage of STN neurons that do not respond to light stimulation (0, 25, 50, 75, and 100%) and of the stimulation gain *k_c_* (2, 6, and 12).

Not surprisingly, the efficacy of the stimulation diminishes as α(*r*) degeneracy increases. While a degeneracy of 50% has an impact on oscillations disruption for *k_c_* = 2 (amplitudes of approximatively 30 sp/s), it can efficiently be tackled by using a larger *k_c_* (*k_c_* = 6 leads to low-amplitude remaining oscillations).

### 3.5. Protocol D: single light source

The size of the STN impedes the use of several light sources. Although holographic techniques could be envisioned (see Section 4 for a discussion about this), it is crucial to test the validity of the proposed closed-loop stimulation signal when only one light source is available for the whole STN population. To this end, we applied the following closed-loop stimulation signal:

(5) $$u(r,t)=u(t)=-k_{c}\int_{\Omega_{1}}\left(z_{1}(r,t)-z_{ref}(r)\right)dr.$$

**Figure 8:**
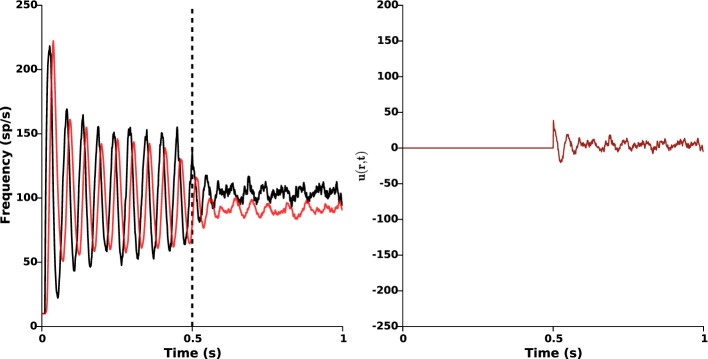
**Single light source**. The same stimulation signal (Equation 5) is provided to all STN neurons (modulo the function α(*r*)), with feedback gain *k_c_* = 6.5 and target reference *z_ref_*(*r*) = 0. Stimulation is ON at time *t* = 0.5 s. **Left:** time evolution of the mean activities of GPe (red) and STN (black). **Right:** time evolution of the closed-loop stimulation signal (Equation 5) (no longer position-dependent). This figure validates the possibility to apply proportional closed-loop stimulation by using a single light source.

This closed-loop stimulation signal can be seen as a spatial average of the original one proposed in Equation (2) over the whole STN population. Like Equation (2), this averaged stimulation signal relies on measurement of STN activity only. Nonetheless, it owns the advantage of being no longer dependent on position: it is taken proportional to the mean STN activity, and is the same for all STN neurons (modulo the function α(*r*)). In this case, a larger feedback gain is required to efficiently disrupt oscillations (*k_c_* = 6.5). The results are presented in Figure 8, and show that proper β-oscillations disruption is achieved. We did not succeed in showing this analytically yet. Surprisingly enough, the required amplitude of stimulation is smaller when a single light source is used (compare the right panels of Figures 5, 8).

### 3.6. Protocol E: effect of acquisition and processing delays

**Figure 9:**
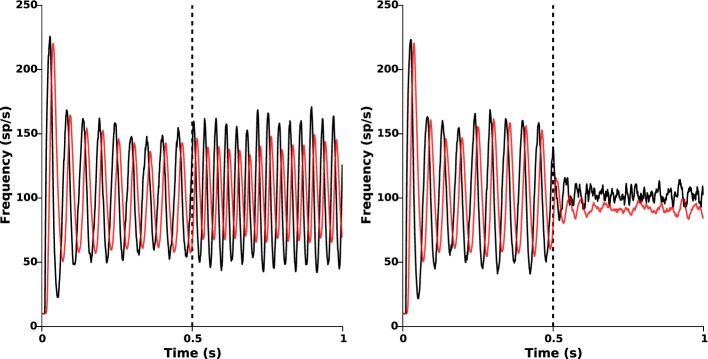
**Effect of acquisition and processing delays**. Time evolution of the mean activities of STN (black) and GPe (red) when the delayed closed-loop stimulation (Equation 6) is applied at time *t* = 0.5 s. **Left:** oscillations disruption is lost for too large delays (*T* = 10 ms). **Right:** β-oscillations disruption is still efficiently achieved for an acquisition and processing delay *T* = 5 ms.

The experimental implementation of the proposed closed-loop stimulation signal in an animal model of Parkinson’s disease would require a real-time estimation of the activity in the STN based on the measurements provided by the recording electrodes. This signal acquisition and its processing require some time, which is likely to induce a significant delay in the applied stimulation signal. In order to test the robustness of the proposed strategy to acquisition and processing delays, we consider the following closed-loop stimulation signal:

(6) $$u(r,t)=-k_{c}(z_{1}(r,t-T)-z_{ref}(r)),$$

where *T* indicates the time needed to acquire and process the STN activity. The results are presented in Figure 9 for a feedback gain *k_c_* = 2.

When *T* is too large (*T* ≥ 10 ms), β-oscillations are no longer disrupted (Figure 9, left panel) and can even be amplified by the stimulation, as already observed in Pasillas-Lépine et al. (2013) in an averaged rate model. Nevertheless, if *T* is reasonably small, oscillations disruption remains satisfactorily achieved (Figure 9, right panel, *T* = 5 ms). This indicates that strong attention should be paid on the code optimization for data processing in view of experimental implementation. Filtering strategies, such as the one presented in Pasillas-Lépine et al. (2013) and a careful choice of the target reference *z_ref_*(*r*) could probably increase robustness to acquisition and processing delays (not implemented here).

**Figure 10:**
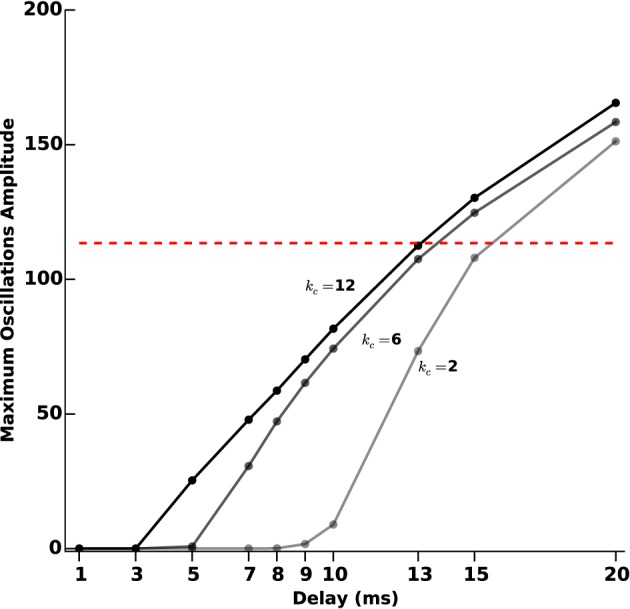
**Oscillations amplitude vs. acquisition and processing delays**. We measure the efficiency of the closed-loop stimulation (Equation 6), which includes processing and acquisition delays *T*, through the amplitude of the remaining oscillations after transients. We consider increasing values of *T* (1, 3, 5, 7, 8, 9, 10, 13, 15, and 20 ms). The three curves correspond to different values of the feedback gain *k_c_* (2, 6, and 12). The darkest line corresponds to the highest value and the lighter to the lowest value. The red dashed line indicates the oscillations maximum amplitude when no stimulation is applied.

In order to assess more finely the impact of acquisition and processing delays *T* and their link with the feedback gain *k_c_*, we tested 10 different delays (1, 3, 5, 7, 8, 9, 10, 13, 15, and 20 ms). For each delay, we measured the oscillations amplitude after transients for different values of *k_c_* (2, 6, and 12), as we did for protocol D. The results are reported in Figure 10.

For small gains (*k_c_* = 2), the model is quite robust to acquisition and processing delays: only delays above 9 ms significantly degrade the performance of the stimulation. This robustness to delays decreases as the feedback gain increases. Depending on the delay value, it may happen that stimulation is even counter-productive, in the sense that sustained oscillations get enhanced rather than disrupted.

## 4. Discussion

In this theoretical work, we have proposed a model of the STN-GPe network able to take into account both temporal and spatial evolutions of electrophysiological activity, and a closed-loop stimulation strategy to suppress sustained oscillations related to Parkinson's disease and test their causality in future experiments with animal models.

Our model is based on delayed neural fields to account for spatiotemporal dynamics. The need to consider spatial heterogeneity in STN and GPe arises from the facts that: (i) the efficiency of DBS requires a fine positioning of the stimulation electrodes (a millimetric scale precision is required, Anheim et al., 2008; Luján et al., 2009), thus indicating a strong position-dependency of the populations sensitivity; (ii) connectivity organization between neurons among a population or between populations cannot be taken into account in averaged models, although some studies underline its crucial role in oscillations onset (Schwab et al., 2013); (iii) the complex functioning of the structures involved, assimilated in Montgomery (2007) to a hierarchical interconnection of multiple nested, loosely coupled oscillators, requires spatially distributed models; (iv) the use of multi-plot electrodes with the most recent technologies permits a spatial resolution below 50 μ m, which cannot be exploited in averaged models; and (v) human recordings indicate a possible role of spatial correlation of STN synchronization in the parkinsonian motor symptoms (Cagnan et al., 2015).

We chose to model this spatiotemporal dynamics of the STN-GPe network by neural fields (Coombes, 2005; Bressloff, 2012). Neural fields were originally introduced by Amari (1977) to model cortical layers. They have been extensively used in the study of many different cortical phenomena such as propagating waves (Cremers and Herz, 2002; Bressloff and Webber, 2012; Meijer and Coombes, 2014), synaptic depression (Bressloff and Kilpatrick, 2011), and neural oscillations (Hutt et al., 2008). As proposed in Holgado et al. (2010) and confirmed in this work, transmission delays between STN and GPe are likely to play a key role in pathological oscillations onset and are thus included in the model. Neural fields affected by axonal propagation and synaptic delays have been studied in Faye and Faugeras (2010); Veltz and Faugeras (2011), which provide the mathematical framework this study relies on. They have been used in (Modolo et al., 2010) for closed-loop stimulation, using a term-cancellation that requires precise knowledge of the dynamics involved, including synaptic weights distributions.

The proposed spatiotemporal STN-GPe model reproduces sustained β-oscillations in a robust way, meaning even for relatively large uncertainty on parameters. We have proposed a closed-loop stimulation policy that provably disrupts sustained oscillations in this spatiotemporal model of the STN-GPe network. Inspired by Pasillas-Lépine et al. (2013), this closed-loop stimulation signal is taken proportional to the STN activity. Assuming that STN neurons have successfully undergone photosensitization, we have formally shown that, provided that no endogenous oscillations take place within the GPe, stabilization of the whole network can always be achieved by this closed-loop stimulation if the feedback gain is taken sufficiently large. This result shows that any oscillations generated within the STN or resulting from a pacemaker effect between STN and GPe populations can be tackled in this model. It is worth stressing that the assumptions of this theoretical result do not rely on the precise knowledge of the parameters and functions involved in the model. No precise information on the activation functions are required: an estimate of their maximum slopes is enough. Similarly, the synaptic weight distributions do not need to be known precisely: all we need to know is whether the intensity of the lateral GPe connections is strong or weak. Also, no knowledge is required on the time constants or on the delays. Finally, no precise tuning of the feedback gain is required: it is enough to pick it sufficiently large. These features make our closed-loop stimulation signal intrinsically robust to parameter uncertainties and promising from an experimental testing perspective. The requirement that lateral synaptic gains of the GPe be low enough is in agreement with the experimental findings of Bar-Gad et al. (2003), where functional correlations between GPe neurons have been reported as either weak or non-existent.

The spatial nature of the model allows to conduct more precise *in silico* experiments exploiting the geometry of STN and GPe and the synaptic distributions of neurons within and between these structures. Our numerical experiments revealed that the model is robust to poor STN photosensitization and that, in case of low photosensitization, a larger feedback gain can be used in order to successfully preserve oscillations disruption.

From an experimental testing in animal models of Parkinson's disease perspective, we stress that the use of optogenetics neuromodulation is crucial since it enables simultaneous neuro-excitation, neuro-inhibition and neural recordings, with biotechnological tools already validated experimentally (Han and Boyden, 2007). Moreover, these numerical findings point toward a necessary trade-off between robustness to poor photosensitization, which is favored by the use of large feedback gains, and robustness to acquisition and processing delays, which is better with small feedback gains. Simulations revealed that the proposed strategy is little robust to acquisition and processing delays if a large feedback gain is used. This issue needs to be carefully taken into account when performing experimentations in animal models of Parkinson's disease. Careful attention should be paid to the optimization of the acquisition algorithms in order to reduce the round-trip-time as much as possible. Future work should be conducted to make the proposed closed-loop stimulation more robust to these delays. This may take the form of a low-pass filter, as in Pasillas-Lépine et al. (2013), or of more advanced strategies inspired from control theory (including predictive features).

For experimental translation, the ability to deliver a space-dependent light for photostimulation is also crucial. Light stimulation is usually delivered through an optical fiber connected to a laser or directly by a light-emitting diode, so that all neurons expressing opsins and receiving photons are simultaneously photo-stimulated, receiving a number of photons depending simply on the output of the fiber, the distance to the fiber and the physical properties of the biological tissue interacting with photons. Patterned illumination constitutes a promising way to illuminate precisely shaped regions of interest, with neighboring neurons receiving potentially different patterns of illumination in intensity and in time (Bovetti and Fellin, 2015). Patterned illumination can be performed by phase modulation of light: liquid crystal spatial light modulators (LC-SLMs) can modulate two-photon light according to arbitrary shapes, thus allowing simultaneous illumination of multiple neurons with different phase maps over time in order to probe neuronal networks with complex spatial and temporal patterns of neuronal activation. For instance, optogenetic neuromodulation with millisecond temporal precision and cellular regulation has been performed in retinal ganglion cells expressing channel-rhodopsins (Reutsky-Gefen et al., 2013). Although we have shown through simulations that proportional stimulation remains efficient even when a single light source is used for the entire controlled population, holographic optogenetics thus constitutes a promising technique to provide precise time and space resolution of the closed-loop stimulation signal.

Future work should deepen the dynamics induced by light stimulation in optogenetics experiments. The model used in this work assumes that the impact of photostimulation is similar to that of a fictitious afferent neural population whose rate and excitatory/inhibitory features can be regulated in real time. In particular, the combined effects of intensity and frequency of light stimulation are not captured by the present model. Tighter modeling could be developed in the future based on existing works such as Nikolic et al. (2009).

Synaptic plasticity and homeostasis are not taken into account in the employed model. Recent studies evoke the role of synaptic plasticity in the long-term effects of DBS (Tass et al., 2012; van Hartevelt et al., 2014). Neural fields models including learning mechanisms have been studied in Galtier et al. (2012); Detorakis and Rougier (2014); Fix (2014) and we believe that the results presented here could constitute a relevant framework to deepen the analysis by including plasticity or homeostasis mechanisms.

Furthermore, the analysis conducted here focuses on the STN-GPe interaction. The theoretical result presented here can be readily adapted to more than two neuronal populations by modifying the synaptic coupling distributions. Consequently, our framework is well fitted to assess the possible role of other brain structures in the pathological oscillations onset.

Finally, as stressed in the introduction, the precise mechanisms of parkinsonian oscillations in deep brain structures is still a matter of debate (Bevan et al., 2002; Lang and Zadikoff, 2005). This paper focuses on one possible mechanism, namely the STN-GPe network acting as a pacemaker. This hypothesis is supported by *in vitro* experiments reported in Plenz and Kital (1999) using mature organotypic cortex-striatum-STN-GPe cultures. To the best of our knowledge, no *in vivo* experiments has yet confirmed or infirmed this hypothesis, although Nambu and Tachibana (2014) underlines the role played by STN-GPe reciprocal connections. We believe that the theoretical insights provided by the model presented here and its associated closed-loop stimulation constitute interesting bases for further experimental investigations in animal models of Parkinson's disease in order to confront this hypothesis with other possible mechanisms, including cortical patterning of STN (Magill et al., 2001) and striatal origins of oscillations (McCarthy et al., 2011).

### Conflict of interest statement

The authors declare that the research was conducted in the absence of any commercial or financial relationships that could be construed as a potential conflict of interest.

## Footnotes

^1^Journal extended version submitted to Biological Cybernetics under the title “A firing-rate regulation strategy for closed-loop deep brain stimulation.”

^2^All these functions are taken as zero outside their domain.

^3^https://github.com/gdetor/neuralfieldDBSmodel.

^4^We call *admissible* any initial condition that lies within the ranges of *S*_1_ and *S*_2_.

## Appendix

### Proof of the theoretical result

A thorough analysis of delayed neural fields has been conducted in Faye and Faugeras (2010); Veltz and Faugeras (2011). In particular, given any continuous initial conditions defined on [−*d_max_*; 0], where *d_max_* is such that *d*_1_(*r*, *r*'), *d*_2_(*r*, *r*') ≤ *d_max_* for all *r*, *r*' ∈ Ω, the solutions *z*_1_ and *z*_2_ are known to exist at all times in the whole domain Ω and to be continuous with respect to time (Faye and Faugeras, 2010, Theorem 3.2.1).

Given any constant inputs *I*_1_(*r*) and *I*_2_(*r*), any equilibrium pattern (*z*_1_^*^, *z*_2_^*^) of the neural fields model (3) is given by the following two implicit equations

(A1a) $$\begin{array}{c} \begin{array}{l} z_{1}^{*}(r)=S_{1}(\sum_{j=1}^{2}\int_{\Omega }w_{1j}(r,r^{\prime })z_{j}^{*}(r^{\prime })dr^{\prime }\\ \;+\;I_{1}(r)-k_{c}\alpha (r)(z_{1}^{*}(r)-z_{ref}(r))) \end{array} \end{array}$$

(A1b) $$\begin{array}{c} z_{2}^{*}(r)=S_{2}\left(\sum_{j=1}^{2}\int_{\Omega }w_{2j}(r,r^{\prime })z_{j}^{*}(r^{\prime })dr^{\prime }+I_{2}(r)\right). \end{array}$$

We start by operating a change of variables in order to rewrite the neural fields (Equation 3) in terms of the difference between the firing rate the resulting equilibrium pattern (*z*_1_^*^, *z*_2_^*^), the existence of which is assumed in the statement. To this end, we let *x*_1_(*r*, *t*): = *z*_1_(*r*, *t*) − *z*_1_^*^(*r*) and *x*_2_(*r*, *t*): = *z*_2_(*r*, *t*) − *z*_2_^*^(*r*). Combining Equation (A1) with Equation (3), the stability of the equilibrium pattern (*x*_1_^*^, *x*_2_^*^) for Equation (3) is equivalent to the stability of the origin for the neural field

(A2a) $$\begin{array}{c} \begin{array}{l} \tau_{1}{\dot{x}}_{1}=-x_{1}+S_{1}(\int_{\Omega }w_{11}(r,r^{\prime })x_{1}(r^{\prime },t-d_{1}(r,r^{\prime }))dr^{\prime }\\ \;+\int_{\Omega }w_{12}(r,r^{\prime })x_{2}(r^{\prime },t-d_{2}(r,r^{\prime }))dr^{\prime }-k_{c}\alpha_{1}(r)x_{1}) \end{array} \end{array}$$

(A2b) $$\begin{array}{c} \begin{array}{l} \tau_{2}{\dot{x}}_{2}=-x_{2}+S_{2}(\int_{\Omega }w_{21}(r,r^{\prime })x_{1}(r^{\prime },t-d_{1}(r,r^{\prime }))dr^{\prime }\\ \;+\int_{\Omega }w_{22}(r,r^{\prime })x_{2}(r^{\prime },t-d_{2}(r,r^{\prime }))dr^{\prime }), \end{array} \end{array}$$

where we omitted the argument (*r*, *t*) of some functions for the sake of notation conciseness and, for each *i* ∈ {1, 2},

(A3) $$\begin{array}{c} \begin{array}{l} S_{i}(x):=S_{i}(x+\sum_{j=1}^{2}\int_{\Omega }w_{ij}(r,r^{\prime })z_{j}^{*}(r))\\ \;-\;S_{i}(\sum_{j=1}^{2}\int_{\Omega }w_{ij}(r,r^{\prime })z_{j}^{*}(r)),\;\forall x\in \mathbb{R}. \end{array} \end{array}$$

Note that 𝒮_i_(0) = 0, which confirms the fact that (Equation A2) has an equilibrium at the origin. In order to proceed to the stability analysis of Equation (A2), we rely on the following technical lemma, which provides a useful upper bound on the time derivative of the Lyapunov-Krasovskii functional proposed in Faye and Faugeras (2010) along the solutions of the system under concern. For the sake of generality, we state it in the case of *n* interconnected neuronal populations. Its proof is omitted but results from rather standard computations.

Lemma 1. *Let* 𝒮_1_, 𝒮_2_ : ℝ → ℝ *be any bounded continuous increasing functions satisfying* |𝒮_*i*_(*x*)| ≤ ℓ_*i*_|*x*| *for all x* ∈ ℝ, *where* ℓ_1_ and ℓ_2_ *denote nonnegative constants. Let* α_1_, α_2_: Ω → ℝ_>0_ *be such that* inf_*r*∈Ω_ α*i*(*r*) > 0. *Consider the function*

$$\begin{array}{l} 𝒱(t)=\frac{1}{2}\sum_{i=1}^{2}\int_{\Omega }\tau_{i}x_{i}{\left(r,t\right)}^{2}dr\\ \;+\int_{\Omega }\sum_{i=1}^{n}\beta_{i}(r)\int_{\Omega }^{}\int_{-d_{i}(r,r^{\prime })}^{0}x_{i}{\left(r^{\prime },t-\theta \right)}^{2}d\theta dr^{\prime }dr, \end{array}$$

*where* β_1_, β_2_ : Ω → ℝ_>0_. *Then, given any k*_1_ ≥ 0, *there exists* γ_1_(*k*_1_) *satisfying* γ_1_(*k*_1_) > 0 *for all k*_1_ > 0 *and* lim_*k*_1_→+∞_γ_1_(*k*_1_) = +∞ *such that, given any functions* λ_*ij*_ : Ω→ℝ_>0_, *the derivative of* 𝒱 *along the solutions of the delayed neural field*

(A4a) $$\begin{array}{c} \begin{array}{l} \tau_{1}{\dot{x}}_{1}(r,t)=-a_{1}x_{1}(r,t)+S_{1}(\sum_{j=1}^{2}\int_{\Omega }w_{1j}(r,r^{\prime })x_{j}(r^{\prime },t\\ \;-\;d_{j}(r,r^{\prime }))dr^{\prime }-\;k_{1}\alpha_{1}(r)x_{1}(r,t)) \end{array} \end{array}$$

(A4b) $$\begin{array}{c} \begin{array}{l} \tau_{2}{\dot{x}}_{2}(r,t)=-a_{2}x_{2}(r,t)+S_{2}\\ \;\left(\sum_{j=1}^{2}\int_{\Omega }w_{2j}(r,r^{\prime })x_{j}(r^{\prime },t-d_{j}(r,r^{\prime }))dr^{\prime }\right) \end{array} \end{array}$$

*satisfies, for any admissible initial conditions*,

$$\begin{array}{l} \dot{𝒱}(t)\leq -\sum_{i=1}^{n}\int_{\Omega }(a_{i}+\gamma_{i}(k_{i})-\int_{\Omega }^{}\beta_{i}(r^{\prime })dr^{\prime }\\ \;-\frac{1}{2}\sum_{j=1}^{n}\frac{{\bar{w}}_{ij}(r)}{\lambda_{ij}(r)})x_{i}{\left(r,t\right)}^{2}dr\\ \;-\sum_{i=1}^{n}\int_{\Omega }\left(\beta_{i}(r)-\frac{1}{2}\sum_{j=1}^{n}\lambda_{ij}(r)\right)\\ \;\int_{\Omega }x_{i}{\left(r^{\prime },t-d_{i}(r,r^{\prime })\right)}^{2}dr^{\prime }dr,\;\forall t\geq 0, \end{array}$$

*where w*_*ij*_ : Ω → ℝ_≥0_ *denote any positive functions satisfying*

(A5) $$\begin{array}{c} {\bar{w}}_{ij}(r)\geq \ell_{i}^{2}\int_{\Omega }w_{ij}{\left(r,r^{\prime }\right)}^{2}dr^{\prime },\;\forall r\in \Omega . \end{array}$$

Note that the influence of the delayed and non-delayed terms in the estimate of $\dot{𝒱}$ can be weighted, to some extent, by the functions λ_*ij*_ which can be arbitrarily tuned.

Remark 1. *We stress that, in the statement of Lemma 1, the functions w_*ij*_ can be taken as close as the functions r* ↦ ℓ_*i*_^2^ ∫_Ω_ *w_ij_*(*r*, *r*')^2^*dr*' *as we want. More precisely, given any function* ε : Ω → ℝ_>0_, *there exists a positive function w_ij_ satisfying (Equation A5) and* $\left|{\bar{w}}_{ij}(r)-\ell_{i}^{2}\int_{\Omega }^{}w_{ij}{\left(r,r^{\prime }\right)}^{2}dr^{\prime }\right|\leq \varepsilon (r)$ *for all r* ∈ Ω. *For instance, the choice* ${\bar{w}}_{ij}(r)=\max\left\{\varepsilon (r);\ell_{i}^{2}\int_{\Omega }^{}w_{ij}{\left(r,r^{\prime }\right)}^{2}dr^{\prime }\right\}$ *fits these requirements.*

Observe that the neural fields (Equation A2) can be written as Equation (A1) by letting α_1_(*r*) = α(*r*) and *a*_1_ = *a*_2_ = 1. Moreover, note that Equation (A3) and the fact that the functions *S_i_* are ℓ_*i*_-Lipschitz ensure that |𝒮_*i*_(*x*)| ≤ ℓ_*i*_|*x*| for all *x* ∈ ℝ. Invoking Lemma 1 it follows that, given any *k_c_* > 0, there exists γ(*k_c_*) > 0 satisfying lim_*k_c_*→+∞_ γ(*k_c_*) = +∞ such that, given any functions β_*i*_, λ_*ij*_:Ω → ℝ_>0_, *i*, *j* ∈ {1,2}, the derivative of the function

$$\begin{array}{l} 𝒱(t)=\frac{1}{2}\sum_{i=1}^{2}\int_{\Omega }\tau_{i}x_{i}{\left(r,t\right)}^{2}dr\\ \;+\int_{\Omega }\sum_{i=1}^{2}\beta_{i}(r)\int_{\Omega }\int_{-d_{i}(r,r^{\prime })}^{0}x_{i}{\left(r^{\prime },t-\theta \right)}^{2}d\theta dr^{\prime }dr, \end{array}$$

along the solutions of Equation (A2) satisfies, for all admissible initial conditions,

$$\begin{array}{l} \dot{𝒱}(t)\leq -\int_{\Omega }(1+\gamma (k_{c})-\frac{{\bar{w}}_{11}(r)}{2\lambda_{11}(r)}-\frac{{\bar{w}}_{12}(r)}{2\lambda_{12}(r)}\\ \;-\int_{\Omega }\beta_{1}(r^{\prime })dr^{\prime })x_{1}{\left(r,t\right)}^{2}dr\\ \;-\int_{\Omega }(1-\frac{{\bar{w}}_{21}(r)}{2\lambda_{21}(r)}-\frac{{\bar{w}}_{22}(r)}{2\lambda_{22}(r)}-\int_{\Omega }\beta_{2}(r^{\prime })dr^{\prime })x_{2}{\left(r,t\right)}^{2}dr\\ \;-\sum_{i=1}^{2}\int_{\Omega }\int_{\Omega }(\beta_{i}(r)-\frac{1}{2}\sum_{j=1}^{2}\lambda_{ji}(r))x_{i}(r^{\prime },t\\ \;-\;d_{j}(r,r^{\prime }){)}^{2}dr^{\prime }dr, \end{array}$$

where the functions *w*_*ij*_ were defined in Equation (A5). The right-hand side of this inequality is a negative definite function in terms of the non-delayed terms provided that, for all *r* ∈ Ω,

(A6a) $$\begin{array}{c} 1+\gamma (k_{c})>\frac{{\bar{w}}_{11}(r)}{2\lambda_{11}(r)}+\frac{{\bar{w}}_{12}(r)}{2\lambda_{12}(r)}+\int_{\Omega }\beta_{1}(r^{\prime })dr^{\prime } \end{array}$$

(A6b) $$\begin{array}{c} \;1>\frac{{\bar{w}}_{21}(r)}{2\lambda_{21}(r)}+\frac{{\bar{w}}_{22}(r)}{2\lambda_{22}(r)}+\int_{\Omega }\beta_{2}(r^{\prime })dr^{\prime } \end{array}$$

(A6C) $$\begin{array}{c} \;2\beta_{1}(r)\geq \lambda_{11}(r)+\lambda_{21}(r) \end{array}$$

(A6d) $$\begin{array}{c} \;2\beta_{2}(r)\geq \lambda_{12}(r)+\lambda_{22}(r). \end{array}$$

Recalling that *W*_22_ = ∫_Ω_ *w*_22_(*r*')*dr*', it can be checked that the following choices satisfy (Equations A6b–d):

$$\begin{array}{l} \lambda_{11}(r)=2{\bar{w}}_{11}(r)\\ \lambda_{12}(r)=\frac{1-{\bar{W}}_{22}}{2\left(2-{\bar{W}}_{22}\right)}{\bar{w}}_{22}(r)\\ \lambda_{21}(r)=\frac{2(3-{\bar{W}}_{22})}{\left(1-{\bar{W}}_{22})(2-{\bar{W}}_{22}\right)}{\bar{w}}_{21}(r)\\ \lambda_{22}(r)=\frac{3-{\bar{W}}_{22}}{2\left(2-{\bar{W}}_{22}\right)}{\bar{w}}_{22}(r)\\ \beta_{1}(r)={\bar{w}}_{11}(r)+\frac{3-{\bar{W}}_{22}}{\left(1-{\bar{W}}_{22})(2-{\bar{W}}_{22}\right)}{\bar{w}}_{21}(r)\\ \beta_{2}(r)=\frac{{\bar{w}}_{22}(r)}{2}. \end{array}$$

In view of Equation (4), Remark 1 ensures that *w*_22_ can be picked in such a way that *W*_22_ = ∫_Ω_ *w*_22_(*r*')*dr*' < 1. Consequently, the functions λ_*ij*_ and β_*i*_ are all positive as required. With these choices, the condition (Equation A6a) boils down to

$$\begin{array}{l} \gamma (k_{c})>\frac{\left(2-{\bar{W}}_{22}){\bar{w}}_{12}(r\right)}{\left(1-{\bar{W}}_{22}){\bar{w}}_{22}(r\right)}+\int_{\Omega }{\bar{w}}_{11}(r^{\prime })dr^{\prime }\\ \;+\;\frac{3-{\bar{W}}_{22}}{\left(1-{\bar{W}}_{22})(2-{\bar{W}}_{22}\right)}\int_{\Omega }{\bar{w}}_{21}(r^{\prime })dr^{\prime }. \end{array}$$

In view of Remark 1 and Equation (A5), a sufficient condition for this to hold is

$$\begin{array}{l} \gamma (k_{c})>\text{​​}\frac{(2-{\bar{W}}_{22})\int_{\Omega }^{}\ell_{1}^{2}w_{12}{\left(r,r^{\prime }\right)}^{2}dr^{\prime }}{\left(1-{\bar{W}}_{22}){\bar{w}}_{22}(r\right)}\text{​}+\text{​}\int_{\Omega }\int_{\Omega }\ell_{1}^{2}w_{11}{\left(r,r^{\prime }\right)}^{2}dr^{\prime }dr\\ \;+\;\frac{3-{\bar{W}}_{22}}{\left(1-{\bar{W}}_{22})(2-{\bar{W}}_{22}\right)}\int_{\Omega }\int_{\Omega }\ell_{2}^{2}w_{21}{\left(r,r^{\prime }\right)}^{2}dr^{\prime }dr^{\prime }, \end{array}$$

Recalling that γ(*k_c_*) can be picked as large as we want provided that *k_c_* is taken sufficiently large, this condition can always be fulfilled. We thus obtain that, for all admissible initial conditions,

$$\dot{V}(t)\leq -\epsilon \int_{\Omega }\left(x_{1}{\left(r,t\right)}^{2}+x_{2}{\left(r,t\right)}^{2}\right)dr,\;\forall t\geq 0.$$

A straightforward adaptation of (Faye and Faugeras, 2010, Theorem 4.2.2) then ensures the existence of positive constants η_1_, η_2_ such that

$$\|x_{1}(\cdot ,t){\|}^{2}+\|x_{2}(\cdot ,t){\|}^{2}\leq \eta_{1}\left(\|\phi_{1}{\|}^{2}+\|\phi_{2}{\|}^{2}\right)e^{-\eta_{2}t},\;\forall t\geq 0,$$

for all admissible initial conditions ϕ_*i*_, *i* ∈ {1,2}, meaning continuous functions from [−*d_max_*;0] to ℝ with bounded *L*_2_-norm over Ω and satisfying *S**_i_* < ϕ_*i*_(*r*) < *S*_*i*_ for all *r* ∈ Ω. The conclusion follows.
